# The influence of exposure to foreign literature on Chinese readers’ out-group attitudes: the sequential mediating role of emotional investment and cultural empathy

**DOI:** 10.3389/fpsyg.2025.1589631

**Published:** 2025-05-30

**Authors:** Zhi Qi, Hong You

**Affiliations:** ^1^Faculty of Education, University of Melbourne, Melbourne, VIC, Australia; ^2^Wuhan Research Institute, Jianghan University, Wuhan, China

**Keywords:** foreign literature, cultural empathy, out-group attitudes, emotional investment, intercultural understanding

## Abstract

**Introduction:**

This study examines the impact of exposure to foreign literature on out-group attitudes among Chinese readers.

**Methods:**

A sequential mediation model is tested, and the effect of exposure to foreign literature on out-group attitudes is mediated by emotional investment and cultural empathy. The study included 799 Chinese readers of foreign literature. Participants’ ages ranged from 19 to 48 years, with a mean age of 34.72 (SD = 5.03). Using a sequential mediation model, we explored how foreign literature might indirectly shape attitudes toward diverse social groups through heightened emotional and empathetic engagement.

**Results:**

The exposure to foreign literature significantly predicts positive out-group attitudes, both directly and indirectly. Specifically, emotional investment in foreign narratives facilitates a personal connection to characters from different backgrounds, enhancing cultural empathy and, in turn, more favorable out-group attitudes.

**Discussion:**

Our findings highlight that foreign literature is a valuable medium for fostering intercultural empathy by providing readers with indirect experiences of diverse cultural perspectives. Additionally, the study underscores the importance of carefully curated literary selections and structured educational approaches to maximize the positive impact of foreign literature on readers’ attitudes. These results contribute to understanding literature’s role in promoting inclusivity, with implications for educational practices that foster empathy and reduce prejudice. Future research should continue to investigate the specific genres, themes, and teaching techniques that most effectively cultivate cultural empathy and open-mindedness in an increasingly globalized society.

## Introduction

In recent years, the role of literature in fostering empathy and intercultural understanding has received growing scholarly interest, particularly in contexts where cultural boundaries are increasingly fluid (Hakemulder, 2000; Kidd and Castano, 2013; Mar et al., 2010). As globalization intensifies cross-cultural interactions, Chinese readers are more frequently exposed to translated literary works from diverse cultures (Chen, 2024). This literary exposure allows them to engage with narratives that present unfamiliar social norms, values, and perspectives (Zhou et al., 2021), which in turn can facilitate perspective-taking and emotional attunement to out-group experiences (Koopman, 2015; Šimenc, 2016).

Narrative fiction has been widely studied for its capacity to elicit empathy by enabling readers to imaginatively simulate the experiences of others (Djikic et al., 2013; Mar and Oatley, 2008). This process often involves emotional investment, whereby readers form affective bonds with characters, even those from markedly different cultural backgrounds (Bal and Veltkamp, 2013). Such emotional engagement has been linked to increased prosocial behavior and reduced prejudice (Sun L., 2023), suggesting that literature can serve as a medium for social transformation (Johnson, 2012; Vezzali et al., 2015).

Cultural empathy, defined as the ability to understand and appreciate cultural differences while maintaining affective sensitivity (Childs, 1999; Connors, 2015; Zhao, 2012), is a relevant and desirable outcome of this engagement (Lin et al., 2018; Teding van Berkhout and Malouff, 2015). While previous studies have emphasized the general impact of fiction on empathy (Bayram and Holmes, 2020), few have empirically examined the specific pathways—such as emotional investment and cultural empathy—that mediate the effect of literary exposure on attitudes toward cultural out-groups, particularly in the Chinese context (Kim, 2022).

To address this gap, the present study tests a sequential mediation model in which exposure to foreign literature influences out-group attitudes through emotional investment and cultural empathy among Chinese readers. By examining this pathway, our study contributes to a more nuanced understanding of the mechanisms through which literature can function as a catalyst for intercultural understanding and prejudice reduction (Blalock et al., 2024; Hoffman and Cleare-Hoffman, 2011). While previous research has demonstrated the potential of literary narratives to foster empathy and improve attitudes toward out-groups, few studies have tested the specific sequential mediation of emotional investment and cultural empathy, particularly among Chinese readers. These insights may inform curriculum development and cultural policy, especially in educational contexts that seek to promote empathy, reduce prejudice, and strengthen intercultural communication in a globalized world.

### Literature review and hypothesis development

Exposure to foreign literature can function as a form of indirect intergroup contact, enabling readers to engage with diverse social perspectives through imagined experiences. According to narrative transportation theory, becoming emotionally and cognitively immersed in fictional stories allows individuals to vicariously inhabit the lives of characters, which can challenge preconceptions and foster empathy (Green and Brock, 2000; Johnson, 2012). Fiction, particularly when it presents out-group characters in humanized and relatable ways, has been shown to enhance intergroup attitudes by promoting perspective-taking and emotional engagement (Kidd and Castano, 2013; Vezzali et al., 2015).

This process is especially salient in literary settings where readers can explore culturally distant experiences without the perceived risks of direct contact. Vezzali et al. (2012) found that identifying with positive out-group characters in fiction can lead to measurable reductions in prejudice, illustrating the potential of literature to act as a mechanism for social inclusion. These findings are supported by broader research in social and media psychology, which suggests that narrative fiction can operate similarly to face-to-face intergroup interactions by enhancing empathy and reducing stereotyping (Batson et al., 2002; Paluck et al., 2021).

Beyond empathy, literary exposure has been associated with cognitive openness and ideological flexibility. Hodson and Busseri (2012) demonstrated that fiction readers tend to score higher on openness to experience, a trait associated with greater cultural tolerance. Similarly, studies indicate that exposure to diverse narratives can disrupt rigid worldviews and foster more nuanced thinking, which supports the development of inclusive attitudes (Appel and Richter, 2007; Koopman, 2015). Importantly, the thematic nature of the literature influences these effects: texts that highlight cooperation, justice, or intercultural dialogue tend to promote prosocial outcomes, while exposure to antagonistic or dehumanizing narratives can reinforce negative stereotypes (Blogowska and Saroglou, 2013).

The relationship between global literary exposure and openness to diversity aligns with cosmopolitanism theory, which holds that engaging with culturally diverse perspectives fosters a sense of global citizenship and reduces ethnocentric bias (Delanty, 2009). As Cleveland and Chang (2009) noted, individuals who regularly consume international media content, particularly literature, exhibit higher levels of intercultural sensitivity and reduced nationalistic attitudes. In this context, foreign literature emerges as a powerful medium that can reshape intergroup perceptions by stimulating emotional resonance and reflective engagement with out-group perspectives (Wang et al., 2022). Based on this theoretical and empirical foundation, we propose the following hypothesis:

*H1*: Exposure to foreign literature (X) directly influences out-group attitudes (Y) among Chinese readers.

### Emotional investment in foreign literature

Engagement with foreign literature often fosters emotional investment among readers, characterized by empathy, emotional resonance, and cultural engagement. This investment is shaped by readers’ capacity to empathize with characters from diverse backgrounds and to emotionally connect with culturally distinct experiences presented in literary works. Empathy plays a pivotal role in this connection, enabling readers to experience the emotions and perspectives of others. For instance, Huan et al. (2023) demonstrated that Chinese university students majoring in English reported heightened empathy after engaging with British literature, indicating that such narratives can enhance emotional engagement and cultural understanding.

Emotional resonance, the affective response elicited by literature, significantly influences readers’ engagement levels. Research indicates that emotional responses to foreign language texts impact readers’ decisions and engagement, highlighting the centrality of cognitive-emotional interaction in literary investment (Liu and Yu, 2022). This interplay between cognition and emotion underscores the importance of selecting literature that evokes emotional responses to deepen readers’ engagement.

Cultural engagement allows readers to immerse themselves in new cultural contexts and emotional norms, fostering an emotional alignment with the foreign culture that enhances investment in the literature. Pugsley (2005) emphasized the significance of cross-cultural exchange in literature, noting that the selection of Australian literature for the Chinese book market underscores the role of cultural import and export in shaping readers’ emotional connections to foreign narratives. Such exchanges facilitate a deeper understanding and appreciation of diverse cultural perspectives, enriching the reading experience.

Educational strategies in foreign literature instruction can effectively boost emotional engagement by making content more relatable. Huan (2023) observed that incorporating empathy-themed excerpts from British and American literature encouraged students to form emotional connections, thereby increasing their investment in the texts. Similarly, pedagogical approaches that emphasize emotional resonance can motivate and engage students, underlining the importance of literature that not only teaches language but also cultivates emotional engagement.

Despite these positive influences, certain challenges may limit emotional investment. Language barriers and cultural differences can complicate readers’ connection to foreign literature, as understanding nuanced cultural contexts and complex language may require additional cognitive effort. Effective pedagogical strategies that bridge these cultural gaps, however, can enhance readers’ emotional connection, enabling them to fully invest in and appreciate foreign literary works. This body of research highlights the significant role of empathy, emotional resonance, and cultural engagement in enhancing Chinese readers’ emotional investment in foreign literature, indicating a layered and nuanced relationship that benefits from educational support and cultural sensitivity. Based on this theoretical and empirical foundation, we propose the following hypothesis:

*H2*: Exposure to foreign literature (X) directly influences Chinese readers’ emotional investment in foreign literature (M1).

### Cultural empathy as an outcome of literary engagement

Cultural empathy, defined as the capacity to understand and appreciate the perspectives and emotions of individuals from different cultural backgrounds, emerges as a significant outcome of engaging with foreign literature. Through immersion in culturally diverse narratives, readers can transcend linguistic and cultural boundaries, fostering deeper connections with characters and contexts beyond their immediate experiences.

In educational contexts, the integration of literature into foreign language instruction has proven effective in enhancing students’ cross-cultural communicative abilities. Jiang and Wang (2018) emphasize that cultural empathy is integral to successful cross-cultural communication, suggesting that foreign language teaching strategies incorporating literature can significantly bolster students’ cultural empathy. Similarly, Sun L. (2023) highlights that pedagogical approaches focusing on empathy and discomfort in foreign language education can cultivate cultural empathy among English as a Foreign Language learners. By exposing students to global human rights themes in literature and art, learners are encouraged to critically engage with diverse perspectives, developing empathy essential for navigating an increasingly interconnected world.

Engagement with multicultural literature has also been shown to foster empathy by allowing readers to connect emotionally with the experiences and struggles of characters from diverse backgrounds. Conrad (2017) found that students reading multicultural literature gained valuable insights into other cultures, which traditional forms of media or internet resources often fail to provide. Keen (2007) discusses how the imaginative role-taking encouraged by literature, especially novels, can significantly enhance readers’ capacity for empathy, a sentiment echoed by many scholars in literary studies.

In a broader intercultural context, empathy is essential for effective communication and understanding between diverse groups. Zhao (2012) introduces the concept of intersubjective empathy, where the act of empathizing is viewed as a two-way, culturally sensitive exchange. This approach positions readers as active participants in intercultural communication, thereby fostering empathy that respects and appreciates cultural differences. Calloway-Thomas (2010) also emphasizes empathy’s geopolitical and cultural dimensions, highlighting its importance in promoting intercultural understanding within a globalized society.

Thus, foreign literature not only enriches readers’ understanding of different cultural narratives but also serves as a powerful medium for developing cultural empathy. This empathy facilitates deeper connections across cultural divides and enhances readers’ ability to appreciate and value the diversity of human experiences. Based on this literature, the third hypothesis is proposed:

*H3*: Exposure to foreign literature (X) directly influences cultural empathy (M2) among Chinese readers.

### Emotional investment and cultural empathy as mediators of out-group attitudes

Emotional investment, fostered through engagement with foreign literature, enables readers to connect deeply with narratives, leading to more empathetic responses toward out-group members. This immersive experience allows individuals to vicariously inhabit the lives of characters from diverse backgrounds, challenging preconceptions and fostering empathy.

Tarrant et al. (2009) demonstrated that participants who exhibited stronger empathy toward out-group members were more likely to hold favorable attitudes when an inclusive social norm was present. This finding aligns with the notion that narrative engagement can reduce prejudice and foster acceptance by allowing individuals to “practice” empathy in a non-threatening context. Further supporting this, Xiao et al. (2021) found that empathy, particularly when accompanied by prosocial emotions, encourages positive attitudes toward helping out-group members. Their study highlighted that emotional investment in narratives featuring out-group experiences can cultivate empathy, leading to increased prosocial behavior. Similarly, Nesdale et al. (2005) observed that fostering empathy through emotionally engaging content helped children develop more positive attitudes toward other ethnicities, especially when empathetic group norms were reinforced. Their research emphasized the role of empathy in shaping children’s ethnic attitudes and promoting inclusivity.

Empathy in narrative engagement is further emphasized as a tool for attitude transformation in diverse settings. Mallan (2013) found that children’s literature, particularly when embedding multicultural perspectives, can foster empathy in young readers, potentially influencing their real-world attitudes toward marginalized groups. Although fictional engagement alone may not guarantee real-world behavior change, the emotional resonance and empathy generated can be a foundation for positive intergroup attitudes. Vorauer and Sasaki (2012) highlighted that the sequential model underscores the cumulative impact of emotional engagement and empathy. Through the emotional investment fostered by foreign literature, readers will likely develop sustained empathy for cultural differences, encouraging them to adopt more positive and tolerant attitudes toward out-groups. This evidence underscores the importance of foreign literature in creating pathways to cultural empathy and improved out-group attitudes. Based on this literature, the fourth hypothesis is proposed:

*H4*: Exposure to foreign literature (X) indirectly influences out-group attitudes (Y), with a serial mediation of emotional investment in foreign literature (M1) and cultural empathy (M2).

**Figure 1:**
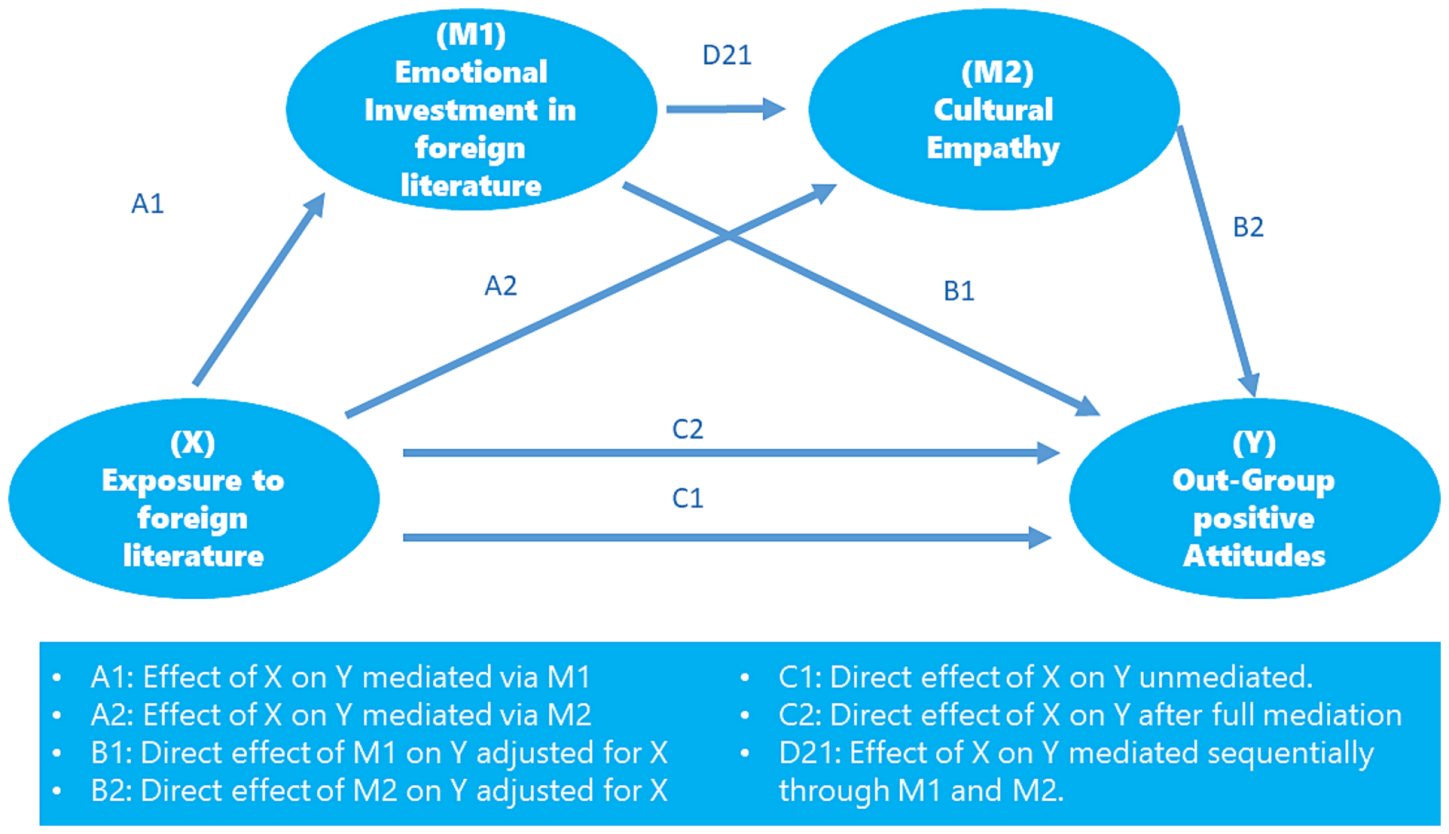
Full model for this research.

The full model tested in this research, which was conceptually developed by the authors based on existing theoretical frameworks, is presented in Figure 1. This proposed model seeks to examine the relationships between supervisor–subordinate guanxi, the components of job embeddedness (fit, links, and sacrifice), and turnover intention.

## Method

### Methodological paradigm

This study is situated within a post-positivist paradigm, which assumes that social phenomena can be studied through empirical observation and analysis, while also acknowledging the influence of contextual and interpretive factors on knowledge production. This paradigm supports the use of structured quantitative methods, such as self-report questionnaires and statistical modeling, to investigate psychological and attitudinal processes. The post-positivist stance allows for the identification of patterns and associations while recognizing that findings are probabilistic rather than deterministic.

### Type of research

This study follows a quantitative, empirical, and explanatory research design. It is quantitative in its use of structured numerical data collected through self-report instruments, and empirical in that it draws on observable data from a large sample of Chinese readers. The study is explanatory in nature, as it seeks to test a theoretically informed model that explains the mechanisms, specifically emotional investment and cultural empathy, through which exposure to foreign literature may influence out-group attitudes.

### Type of study

The study employed a cross-sectional, correlational design. Data were collected at a single point in time using an online survey to examine the associations among exposure to foreign literature, emotional investment, cultural empathy, and out-group attitudes. This design allows for the testing of theoretically driven mediation models, while acknowledging the limitations inherent to non-longitudinal data.

### Participants

The study included 799 Chinese readers of foreign literature. Participants’ ages ranged from 19 to 48 years, with a mean age of 34.72 (SD = 5.03). They reported an average of 9.94 years (SD = 5.39) of experience reading foreign literature, with individual experience spanning from less than a year to a maximum of 38 years. Regarding reading frequency over the last quarter, most reported reading one to three foreign books. Specifically, 256 participants (32.0%) read one book, 181 (22.7%) read two books, and 171 (21.4%) read slightly more than two (recorded as 2.1 on average). Fewer participants reported higher frequencies, with 133 (16.6%) reading three books, 46 (5.8%) reading four, 4 (0.5%) reading five, and only 8 (1.0%) reading ten books. The sample included a slightly higher proportion of female participants, with 452 (56.6%) identifying as female and 347 (43.4%) as male. In terms of educational level, 336 participants (42.1%) held a Master’s degree or Ph.D., 225 (28.2%) had college-level education, 109 (13.6%) had vocational training, and 81 (10.1%) had completed primary education. An additional 34 participants (4.3%) indicated other educational backgrounds. Fourteen participants (1.8%) did not report their educational level. The sample included diverse professions, with participants spanning sectors that commonly engage with foreign literature for professional and personal growth. A substantial portion of the sample comprised individuals in education and academia (27.4%) and university or graduate students (27.3%), groups likely to read foreign literature for academic purposes and personal interest. Corporate and business professionals comprised another significant segment (27.0%), reflecting the demand for global cultural awareness in the business sector. Smaller yet notable representations included media and publishing professionals (6.3%) and those in technology and engineering (3.3%), sectors where exposure to global perspectives is often valued. Government and international relations professionals (2.8%) were also present, likely reflecting interest in foreign literature for cultural and diplomatic insights. Additionally, the sample included smaller groups from social work (0.8%), healthcare (1.1%), art and creative professions (1.5%), and tourism (0.9%)—an additional 1.8% identified with other fields.

### Procedure

This study received ethical approval from the Jianghan University’s Ethical Committee, with Approval protocol number WRI-5671-2023, ensuring compliance with research standards and participant protection. All procedures adhered to the principles outlined in the Declaration of Helsinki and its subsequent amendments. Participants were fully informed about the study’s purpose, procedures, and their rights before participating. Informed consent was obtained electronically from all participants before they engaged with the survey, with assurances of confidentiality and anonymity. Participation was entirely voluntary, and participants were informed that they could withdraw from the survey at any time without penalty.

The study employed a cross-sectional survey design to test a sequential mediation model in which exposure to foreign literature influences out-group attitudes among Chinese readers, through the mediating roles of emotional investment and cultural empathy. The survey was disseminated through various strategic channels to ensure a broad reach among individuals actively reading foreign literature. To connect with readers with specific cultural and literary interests, we partnered with Chinese cultural institutions, book clubs, and literary societies that promote foreign literature. Additionally, the survey was shared on prominent digital reading platforms, including Douban and WeChat reading groups, where communities regularly engage in discussions on foreign books and literature. Social media platforms, such as WeChat, Weibo, and Xiaohongshu (Little Red Book), were also utilized to circulate the survey, allowing for targeted sharing among potential participants interested in foreign cultural content. Finally, invitations to participate in the survey were sent to university departments focusing on foreign languages, literature, and cultural studies. This provided access to a diverse audience of students and professionals in academia. The survey was conducted using a commercial tool (Qualtrics), allowing for secure and efficient data collection and providing participants with a seamless and user-friendly experience. The online survey included a structured questionnaire composed of sections on demographics, reading habits, exposure to foreign literature, and various scales assessing cultural empathy and out-group attitudes. To protect participant data, measures were implemented in line with industry standards. The Qualtrics platform employed encryption and secure data storage, safeguarding responses’ confidentiality and ensuring that authorized researchers could only access data. No identifiable information was collected, and data were anonymized before analysis to protect participant privacy further.

### Instruments

The study assessed four key variables: exposure to foreign literature (X), emotional investment in foreign literature (M1), cultural empathy (M2), and out-group positive attitudes (Y), using multiple survey items for each construct.

*Exposure to foreign literature* was measured through two items designed to capture the frequency and duration of participants’ engagement with foreign literary works. Participants were asked, “For how many years have you been reading foreign literature (novels, short stories, poems, etc.)?” and “On average, how much time per week do you spend reading foreign literature?” to assess the breadth and depth of their reading habits. Responses were provided on a 5-point scale ranging from “None” to “A lot.” Additionally, familiarity with different genres of foreign literature was assessed using a 5-point scale, with items referring to specific genres such as Western novels, European poetry, Latin American magical realism, Japanese manga and anime, and Indian epics. Our measure did not differentiate between literature read in the original language and translated versions; thus, participants may have reported engagement with either form, depending on their linguistic background. The exposure to foreign literature scale included two items assessing reading frequency and duration. As it contained only two items, internal consistency was evaluated using the Pearson correlation coefficient (r = 0.58), which indicated an acceptable level of inter-item association.

*Emotional investment in foreign literature* was assessed using a 10-item scale developed by the authors for the purposes of this study. The scale was designed to measure the affective engagement of readers with foreign literary texts, including dimensions such as emotional immersion, identification with characters, and the lingering emotional impact of narratives. Items were written to reflect readers’ personal involvement in the emotional worlds of the stories they read (e.g., “I become deeply immersed in the emotional experiences of characters in foreign novels” and “The emotional journeys portrayed in foreign narratives tend to linger in my mind long after I’ve finished reading”). Participants rated each item on a 5-point Likert scale, ranging from 1 (“strongly disagree”) to 5 (“strongly agree”). This measure aimed to capture how personally invested readers felt in the emotional content and characters within foreign literary works. Cronbach’s Alpha for this study was 0.78.

### Scale adaptation

Both the Cross-Cultural Empathy Scale and the Intergroup Attitudes Scale were originally developed in English and adapted for use with Chinese participants. The adaptation process involved translation, cultural contextualization, and item review. First, the items were translated into Chinese by a bilingual expert in psychology and literature. A back-translation was then conducted by an independent translator unfamiliar with the original versions. Discrepancies were resolved through discussion among the research team to ensure semantic and conceptual equivalence. In addition, minor wording adjustments were made to ensure cultural relevance and comprehension. No items were removed from the original scales.

*Cultural empathy* was measured using an adapted version of the Cross-Cultural Empathy Scale (Wang et al., 2003), which consists of six items designed to assess participants’ empathic understanding and connection with individuals from other cultures. Participants rated each statement on a 5-point Likert scale, from “strongly disagree” to “strongly agree.” Sample items included “I can understand the reasons behind out-group members’ behaviors” and “I feel connected to people from different cultural backgrounds.” This measure focused on capturing the participants’ capacity to empathize with and relate emotionally to individuals from diverse cultural backgrounds. Cronbach’s Alpha for this study was 0.81.

*Out-group positive attitudes* were assessed with an adapted version of the Intergroup Attitudes Scale (Stephan et al., 1999), which consists of seven items measuring positive perceptions and openness toward people from other cultures. Responses were given on a 5-point Likert scale, ranging from “strongly disagree” to “strongly agree.” Items in this scale included “I have positive feelings towards people from other cultures” and “I would be willing to cooperate with someone from a different cultural background.” This measure evaluated the extent to which participants held favorable attitudes, comfort, and willingness to interact with individuals from culturally diverse backgrounds. Cronbach’s Alpha for this study was 0.79.

### Conceptual model

The sequential mediation model tested in this study was developed by the authors, based on theoretical and empirical research linking narrative engagement, emotional investment, cultural empathy, and intergroup attitudes (Green and Brock, 2000; Kidd and Castano, 2013; Vezzali et al., 2015). While the individual relationships among these constructs have been previously examined, their integration into a unified, sequential model had not been empirically tested within this specific context. The model hypothesizes that exposure to foreign literature increases emotional investment in narratives, which in turn enhances cultural empathy and ultimately fosters more positive attitudes toward out-groups. This framework was evaluated using regression-based mediation analysis (PROCESS macro, Model 6), and demonstrated strong theoretical coherence and statistically significant effects across all hypothesized paths. However, it is important to note that the model has not undergone formal validation through techniques such as confirmatory factor analysis or structural equation modeling, which should be addressed in future research.

### Data analyses

All analyses were conducted using SPSS version 29.0, with PROCESS version 4.2 by Hayes for mediation and moderation testing. Descriptive analyses were initially conducted to assess each primary variable’s mean and standard deviation: exposure to foreign literature, emotional investment in foreign literature, cultural empathy, and out-group positive attitudes. Pearson correlation coefficients were calculated to examine the bivariate relationships among these variables. PROCESS Model 6 was employed to evaluate the serial mediation effects for hypothesis testing. Specifically, the model tested whether exposure to foreign literature (X) influences out-group positive attitudes (Y) directly and indirectly through two mediators: emotional investment in foreign literature (M1) and cultural empathy (M2). Bootstrapping procedures with 5,000 samples were utilized to generate bias-corrected confidence intervals for the indirect effects. Statistical significance for each pathway was determined based on whether the 95% confidence intervals excluded zero. The analysis followed a structured approach to assess each hypothesized relationship in sequence, beginning with the direct effects of exposure on out-group attitudes, emotional investment, and cultural empathy, and subsequently examining the hypothesized mediation paths.

## Results

### Descriptive analyses and correlation matrix

The descriptive statistics for each variable, including exposure to foreign literature, emotional investment in foreign literature, cultural empathy, and out-group positive attitudes, indicate that participants, on average, showed moderate levels across all measures, with consistent sample sizes. The measures show a moderate variability level, suggesting diversity in responses. The correlation matrix reveals significant associations among the study variables. Exposure to foreign literature was positively correlated with emotional investment in foreign literature and cultural empathy, indicating that individuals with higher levels of exposure also tended to report greater emotional and empathetic engagement. Additionally, cultural empathy is significantly correlated with positive out-group attitudes, supporting that empathy toward other cultures is linked with favorable attitudes toward out-groups. The positive associations between these variables support the hypothesized relationships in the model.

Table 1 summarizes these descriptive statistics and correlations.

**Table 1:** Descriptive statistics and correlations.

Variable	Mean	SD	1	2	3
1. Exposure to Foreign Literature	31.98	0.56	–		
2. Emotional Investment in Foreign Literature	37.45	0.66	0.651**	–	
3. Cultural Empathy	28.75	0.85	0.351**	0.307**	–
4. Out-Group Positive Attitudes	29.09	0.66	0.381**	0.221**	0.471**

*N* = 799. SD, Standard Deviation. **p < 0.001.

### Hypotheses testing

#### Hypothesis 1 – direct effect on out-group attitudes

Exposure to foreign literature significantly predicted out-group attitudes, b = 0.3632, SE = 0.0478, *t* (795) = 7.60, *p* < 0.001, 95% CI [0.2694, 0.4570], *β* = 0.3079. The model explained 28.1% of the variance in out-group attitudes, *R*^2^ = 0.2809.

#### Hypothesis 2 – direct effect on emotional investment

Exposure to foreign literature significantly predicted emotional investment, b = 0.7588, SE = 0.0314, *t* (797) = 24.14, *p* < 0.001, 95% CI [0.6972, 0.8204], *β* = 0.6506. This model explained 42.3% of the variance in emotional investment, *R*^2^ = 0.4233.

#### Hypothesis 3 – direct effect on cultural empathy

Exposure to foreign literature significantly predicted cultural empathy, b = 0.3974, SE = 0.0658, *t* (796) = 6.04, *p* < 0.001, 95% CI [0.2683, 0.5265], *β* = 0.2624. Emotional investment was also a significant predictor of cultural empathy, b = 0.1771, SE = 0.0564, *t* (796) = 3.14, *p* = 0.002, 95% CI [0.0664, 0.2878], *β* = 0.1364. The model explained 13.4% of the variance in cultural empathy, *R*^2^ = 0.1341. The regression analysis results are also displayed in Tables 2, 3.

**Table 2:** Model summary for each outcome variable.

Outcome variable	*R*	*R* ^2^	MSE	*F*	df₁	df₂	*p*
Emotional Investment (M1)	0.651	0.423	0.2521	585.05	1	797	< 0.001
Cultural Empathy (M2)	0.366	0.134	0.6391	61.62	2	796	< 0.001
Out-Group Attitudes (Y)	0.530	0.281	0.3226	103.53	3	795	< 0.001
Total Effect Model	0.381	0.145	0.3826	135.21	1	797	< 0.001

*R* = multiple correlation coefficient; *R*^2^ = coefficient of determination; MSE, mean squared error.

**Table 3:** Regression coefficients for all outcome variables.

Outcome variable	Predictor	*b*	SE	*t*	95% CI (LL, UL)	β
Emotional investment	Constant	13.183	0.102	129.37	[13.183, 15.183]	–
	Exposure to Foreign Lit.	0.759	0.031	24.14	[0.697, 0.820]	0.651
Cultural empathy	Constant	0.941	0.179	5.26	[0.591, 1.291]	–
	Exposure to Foreign Lit.	0.397	0.066	6.04	[0.268, 0.527]	0.262
	Emotional Investment	0.177	0.056	3.14	[0.066, 0.288]	0.136
Out-group attitudes	Constant	12.453	0.129	96.55	[12.200, 12.706]	–
	Exposure to Foreign Lit.	0.363	0.048	7.60	[0.269, 0.457]	0.308
	Emotional Investment	−0.102	0.040	−2.53	[−0.181, −0.023]	−0.101
	Cultural Empathy	0.307	0.025	12.20	[0.258, 0.357]	0.394

CI, confidence interval; β, standardized coefficient.

#### Serial mediation of emotional investment and cultural empathy (hypothesis 4)

Hypothesis 4 proposed a serial mediation model in which exposure to foreign literature influences out-group attitudes indirectly through emotional investment and cultural empathy. The results of the regression-based path analysis supported this hypothesis, revealing a significant total indirect effect, β = 0.0861, SE = 0.0355, 95% CI [0.0177, 0.1579], indicating that psychological engagement variables partially mediate the relationship between exposure and out-group attitudes.

Three indirect pathways were examined. The indirect effect of exposure to foreign literature on out-group attitudes through emotional investment alone (X → M1 → Y) was significant and negative, β = −0.0773, SE = 0.0296, 95% CI [−0.1362, −0.0187], suggesting that emotional investment, when not accompanied by cultural empathy, may not necessarily translate into more favorable out-group attitudes. In contrast, the indirect effect through cultural empathy alone (X → M2 → Y) was positive and significant, β = 0.1221, SE = 0.0232, 95% CI [0.0770, 0.1692], indicating that readers with higher exposure to foreign literature tend to develop greater cultural empathy, which in turn is associated with improved attitudes toward out-groups.

The serial mediation pathway (X → M1 → M2 → Y), in which emotional investment enhances cultural empathy, which then leads to more favorable out-group attitudes, was also significant, β = 0.0413, SE = 0.0152, 95% CI [0.0130, 0.0725]. This confirms the theoretical model suggesting that foreign literature promotes attitudinal change through affective involvement and deepening readers’ cultural understanding. These results support a partial serial mediation and underscore the relevance of emotional and cultural processes in shaping readers’ social perceptions. The detailed coefficients for each indirect effect are presented in Table 4.

**Table 4:** Indirect effects of exposure to foreign literature on out-group attitudes.

Pathway	Effect (β)	Boot SE	95% CI (LL, UL)
Total indirect effect	0.0861	0.0355	[0.0177, 0.1579]
Exposure → Emotional investment → Out-group attitudes	−0.0773	0.0296	[−0.1362, −0.0187]
Exposure → Cultural empathy → Out-group attitudes	0.1221	0.0232	[0.0770, 0.1692]
Exposure → Emotional investment → Cultural empathy → Out-group attitudes	0.0413	0.0152	[0.0130, 0.0725]

All indirect effects were derived using 5,000 bootstrap samples. CI, confidence interval; Boot SE, bootstrap standard error. An indirect effect is considered statistically significant if the 95% CI does not include zero.

**Figure 2:**
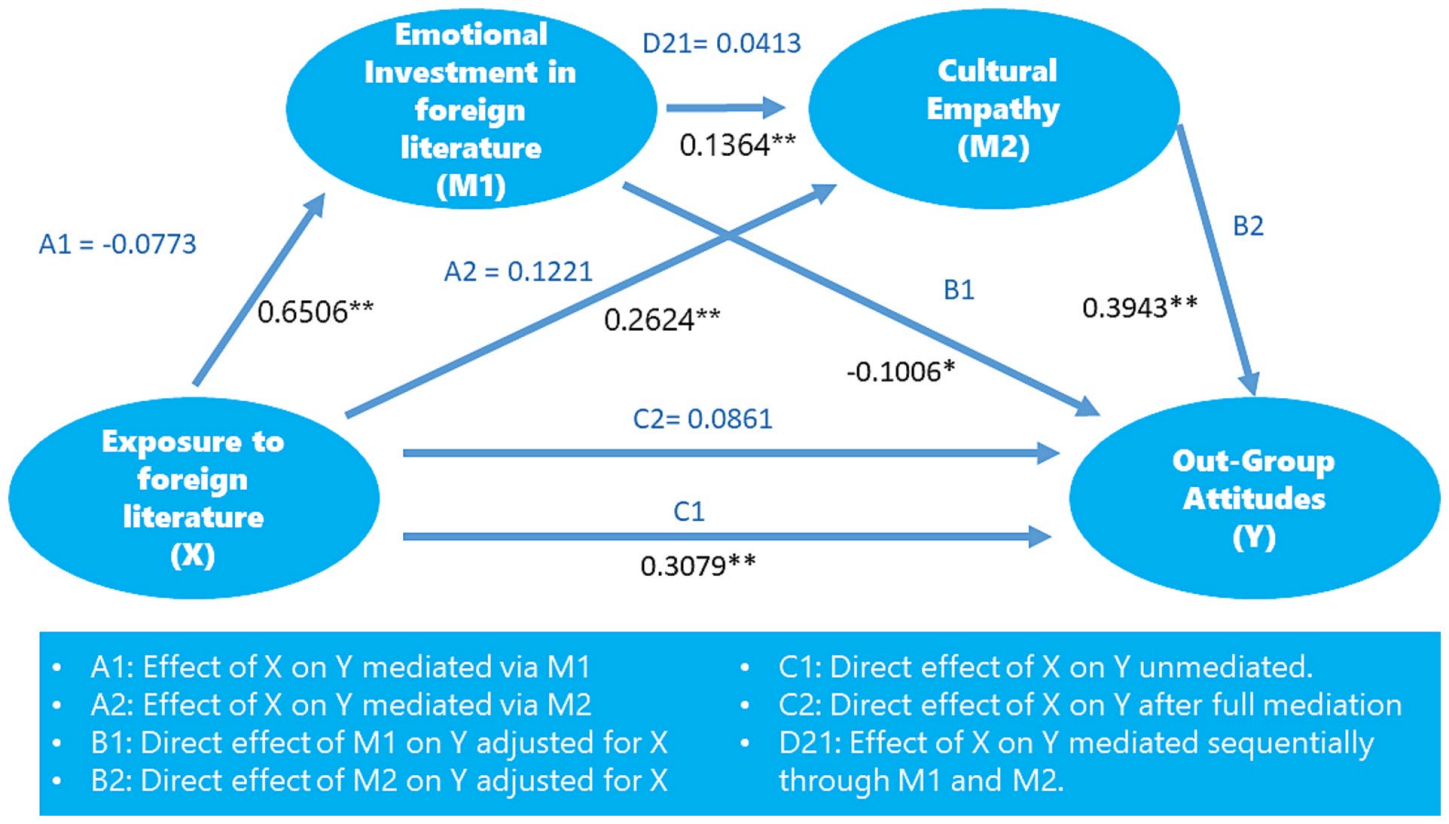
Full model of the study. Standardized direct effects are in black, and Unstandardized indirect effects are in blue. ** *p <* 0.001; *p < 0.05.

Figure 2 displays the full study model with the standardized direct and unstandardized indirect effects.

The figure illustrates the hypothesized relationships between exposure to foreign literature, emotional investment, cultural empathy, and out-group positive attitudes, along with the estimated path coefficients. Exposure to foreign literature (X) has direct and indirect effects on out-group positive attitudes (Y). The direct path from exposure to out-group attitudes suggests that greater exposure to foreign literature is associated with more favorable attitudes toward out-groups. The figure also highlights two mediating pathways: emotional investment in foreign literature (M1) and cultural empathy (M2). Exposure to foreign literature positively influences emotional investment, impacting cultural empathy. Both mediators contribute significantly to the indirect effects of exposure on out-group attitudes. The serial mediation pathway—exposure to emotional investment, cultural empathy, and out-group attitudes—supports the hypothesized model, indicating that readers who engage deeply with foreign literature are more likely to develop empathy for other cultures and, consequently, display more positive attitudes toward out-groups. The path coefficients for each relationship are provided in the figure, with statistically significant paths marked, affirming the robustness of the hypothesized relationships. This visual representation underscores the importance of emotional and empathetic engagement as mechanisms through which exposure to foreign literature can enhance intercultural understanding and acceptance.

## Discussion

The present study tested a sequential mediation model in which exposure to foreign literature was hypothesized to influence Chinese readers’ out-group attitudes through emotional investment and cultural empathy. The findings largely supported the model. Specifically, exposure to foreign literature was positively associated with more favorable out-group attitudes, aligning with prior work suggesting that literary engagement can serve as a form of indirect intergroup contact. Our data confirm this association in a non-Western context, providing empirical support for the role of narrative exposure in shaping social attitudes beyond Western populations.

This effect is especially relevant in the Chinese context, where state-regulated publishing practices and cultural gatekeeping shape exposure to foreign literature. The significant direct relationship between exposure and out-group attitudes suggests that, despite these structural filters, foreign literary narratives can influence readers’ social perceptions. While prior literature has discussed the potential of literary texts to foster empathy (Green and Brock, 2000; Kidd and Castano, 2013), our study advances this line of research by demonstrating that emotional and cognitive mechanisms play distinct and sequential roles in shaping attitudes. The statistical mediation analysis reveals that while emotional investment contributes to attitudinal outcomes, the development of cultural empathy ultimately accounts for improved perceptions of out-groups.

The results confirmed that exposure to foreign literature significantly predicted emotional investment, supporting the view that repeated engagement with culturally diverse narratives enhances readers’ affective involvement with texts. Readers with higher exposure levels were likelier to report sustained emotional engagement, suggesting that familiarity with foreign cultural material may facilitate a sense of narrative immersion or identification. These findings offer empirical support for claims in literary psychology regarding the immersive nature of fiction, but extend them by showing how this emotional connection emerges specifically in the context of intercultural reading.

Interestingly, the negative direct effect of emotional investment on out-group attitudes complicates the assumption that emotional resonance alone leads to more inclusive views. This suggests that affective engagement may generate ambivalent or adverse responses without cognitive mechanisms such as cultural understanding. For example, emotionally intense narratives may provoke discomfort, confusion, or even defensiveness if the cultural content is perceived as morally dissonant or socially distant. These findings are consistent with emerging work on the dual effects of narrative engagement, highlighting that emotional arousal can increase empathy but also risk reinforcing bias depending on how the reader processes the experience (Keen, 2007; Vorauer and Sasaki, 2012). Importantly, the positive indirect effect observed through the sequential pathway (exposure → emotional investment → cultural empathy → out-group attitudes) reinforces the idea that emotional investment contributes positively to attitudinal change when it facilitates the development of cultural empathy.

The data also supported the hypothesis that exposure to foreign literature predicts greater cultural empathy among Chinese readers. This relationship reinforces the idea that regular engagement with culturally diverse narratives can enhance readers’ capacity to understand and appreciate perspectives different from their own. Unlike emotional investment, cultural empathy in this model was consistently associated with more positive out-group attitudes, suggesting that it may be the central psychological mechanism linking literary exposure to social perception.

This finding extends previous research on narrative empathy by demonstrating that cultural empathy is not merely an emotional reaction to characters but a broader disposition toward intercultural understanding that can be cultivated through literary experience. Whereas emotional investment may elicit strong but ambivalent reactions, cultural empathy provides the interpretive and reflective framework necessary for readers to process unfamiliar cultural material in ways that support inclusivity. Our results suggest that foreign literature contributes to this process by offering readers sustained exposure to alternative worldviews, encouraging perspective-taking, and reducing ethnocentric bias. Importantly, these effects were observed where access to foreign literature is often mediated by selective translation and cultural gatekeeping, indicating the robustness of literature’s potential to foster empathy even within constrained environments.

The sequential mediation analysis confirmed that exposure to foreign literature influences out-group attitudes directly and indirectly through the combined effects of emotional investment and cultural empathy. While emotional investment alone had a small but significant negative effect on out-group attitudes, the full sequential pathway (exposure → emotional investment → cultural empathy → out-group attitudes) was positive and statistically significant. This finding aligns with previous research suggesting that emotional engagement with narrative texts can foster empathy and influence social attitudes (Kidd and Castano, 2013; Mar and Oatley, 2008), but our results nuance this view by showing that emotional investment may lead to more favorable intergroup perceptions only when accompanied by cognitive-affective understanding. These findings support recent evidence that empathy-related outcomes depend not only on emotional arousal but also on how readers interpret and internalize the cultural meaning of narratives (Keen, 2007; Vorauer and Sasaki, 2012).

The quality and mode of translation significantly impact readers’ emotional connections to foreign literature. Accurate and culturally sensitive translations can facilitate understanding and empathy, enhancing the emotional resonance of the text and enabling deeper engagement with its characters and cultural themes (Kwok, 2016). Conversely, poor or overly literal translations may hinder comprehension and reduce emotional investment by distorting cultural references or narrative tone. In the present study, participants reported engaging with foreign literature without specifying whether texts were read in the original language or translation. Both forms of exposure were likely included, depending on individual language proficiency. Future research could examine whether the language of literary exposure moderates the psychological pathways identified here, as reading in translation versus the source language may involve distinct cognitive and emotional processes (Sun X., 2023; Wang, 2013).

Taken together, the findings offer empirical support for the role of foreign literature in promoting intercultural understanding. While narrative exposure fosters affective engagement, the transition from emotional resonance to cultural empathy appears to drive meaningful changes in attitudes toward out-groups. This observation is consistent with literature on perspective-taking and narrative empathy as mechanisms for attitude change (Batson et al., 2002; Vezzali et al., 2015). Our study contributes to this field by providing evidence from a non-Western sample and highlighting the importance of serial psychological processes. These results suggest that literary interventions—particularly in educational or cultural settings—may benefit from selecting works that evoke emotional responses and provide sufficient cultural context to support reflective engagement. Future studies should investigate the narrative features, pedagogical strategies, and reader variables that most effectively foster cultural empathy and reduce intergroup bias in diverse populations.

### Limitations of the present study

While this study offers valuable insights into the relationship between exposure to foreign literature, cultural empathy, and out-group attitudes among Chinese readers, several limitations should be acknowledged. First, the cross-sectional design limits causal inferences regarding the directionality of observed associations. Although exposure to foreign literature was significantly related to emotional and attitudinal outcomes, the data do not allow for definitive conclusions about temporal or causal sequencing. Future research employing longitudinal or experimental designs would be better positioned to assess whether sustained engagement with foreign literature actively fosters increases in empathy or shifts in intergroup attitudes over time.

Second, the study relied on self-report measures, which are inherently susceptible to social desirability bias and inaccuracies in self-assessment. Participants may have overreported empathy or positive attitudes toward out-groups due to perceived social expectations. Moreover, self-reported engagement with foreign literature may not accurately reflect that exposure’s quality, depth, or nature. To address these issues, future studies could incorporate behavioral assessments, implicit measures, or observational data to more accurately capture literary engagement’s cognitive and affective effects.

Third, the study did not differentiate between foreign literature read in translation and that read in the original language. While “foreign literature” was intentionally broad to accommodate a wide range of reading experiences, this lack of specification may obscure potential emotional or cognitive impact differences. As translation quality and linguistic familiarity can shape readers’ interpretive and empathic responses, future research would benefit from disaggregating these forms of exposure to explore whether the language of reading moderates the pathways observed in this study.

Fourth, although the sample was heterogeneous regarding age, education, and occupation, it was limited to individuals with internet access and familiarity with online survey platforms. This may have excluded important subgroups, such as older adults or rural populations, who may engage with literature through different channels or have distinct reading practices. Expanding data collection methods to include offline recruitment or partnerships with community centers, bookstores, or libraries could improve future studies’ representativeness and ecological validity.

Fifth, the conceptual model proposed and tested in this study was developed by the authors to integrate theoretically supported relationships among narrative engagement, emotional investment, cultural empathy, and intergroup attitudes. Although the model demonstrated strong theoretical coherence and statistically significant paths, it has not been formally validated using structural equation modeling or confirmatory factor analysis. This lack of formal validation limits the assessment of the model’s structural integrity and potential generalizability. Future research should aim to replicate the model across independent samples and apply advanced validation techniques to strengthen its empirical foundation.

Finally, the study’s focus on Chinese readers may limit the generalizability of the findings to other national or cultural contexts. Responses to foreign literature are likely shaped by readers’ cultural schemas, educational backgrounds, and broader media environments. Comparative studies across cultural settings could provide more comprehensive insights into how literature functions as a medium for empathy and intergroup understanding in diverse sociocultural contexts (Chu-Fuluifaga and Reynolds, 2023). Such work would help clarify the extent to which the mechanisms identified here are culturally specific or globally applicable.

### Suggestions for foreign literature teachers

While exposure to foreign literature has the potential to positively influence attitudes toward out-groups by fostering empathy and cultural understanding, educators must also be attentive to the complex and sometimes contradictory effects that literary narratives may produce. Not all foreign texts promote inclusivity; on the contrary, some may inadvertently reinforce stereotypes or cultural biases, especially if they portray out-groups in reductive or negative ways. As such, literature instructors have a critical role in curating texts and designing pedagogical strategies that maximize the development of empathy while mitigating potential misinterpretations (Bobkina et al., 2021).

An effective pedagogical approach involves structured engagement before, during, and after reading, designed to cultivate emotional resonance and cultural insight. Pre-reading activities introducing historical, political, or cultural contexts—alongside empathy-focused prompts—can prepare students to approach unfamiliar material with openness and sensitivity (Arabi, 2012). During-reading discussions enable students to share reactions, clarify misunderstandings, and interpret culturally rich content collaboratively. Post-reading reflections, including guided discussions or analytical responses, allow students to articulate how the texts have challenged or deepened their assumptions about others (Davletbaeva et al., 2016).

Empathy development can be further supported through targeted assignments that prompt students to explore characters’ internal experiences and cultural positioning. Reflective journals, character diaries, or empathy-based essays allow students to engage with both the affective and cognitive dimensions of literary experience (Knutson, 2000). Recent studies suggest that students’ written reflections on foreign texts often reveal increased awareness of cultural complexity and shared humanity, particularly when instructors encourage attention to underlying emotional themes and cultural context (Huan et al., 2023). Additionally, integrating cultural knowledge with language instruction can enhance intercultural competence. When students engage with texts that explore unfamiliar norms, values, and social roles, they develop a more nuanced and communicatively effective understanding of cultural difference (Belyakov et al., 2023; Shi et al., 2016).

Careful text selection is especially important in avoiding simplified or stereotypical out-group portrayals. Educators are encouraged to prioritize works that offer multidimensional and realistic representations, which can challenge students’ preconceptions and foster more inclusive worldviews (Camicia, 2007; Michaels, 2003). Paired with reflective assignments—such as personal response papers or guided journaling—these texts can promote critical thinking and help students recognize and adjust their implicit biases over time (Komarova et al., 2020; Perry, 2023). This process may also strengthen students’ cognitive flexibility and perspective-taking capacities, which are central to long-term empathy development.

Instructors should consider providing supplementary materials such as glossaries, cultural notes, or interpretive prompts to further support student engagement, particularly in contexts where language and cultural barriers may present obstacles. These resources can enhance accessibility and reduce cognitive overload, allowing students to focus more fully on the human dimensions of the narrative. For bilingual learners, reading foreign literature in the original language may amplify empathy by preserving the cultural and emotional nuances embedded in the source text (Wu et al., 2020).

Overall, this study’s findings underscore the potential of foreign literature to serve as a meaningful tool for fostering cultural empathy and improving attitudes toward diverse social groups. By engaging with narratives that represent multiple perspectives, students develop the emotional and cognitive foundations necessary for understanding cultural differences. The sequential mediation model tested here highlights emotional investment and cultural empathy as key psychological pathways through which literary exposure shapes social attitudes. This suggests that indirect contact through literature can function as a valuable mechanism for reducing prejudice and broadening cultural awareness.

To realize this potential, instructors must adopt pedagogical strategies that promote reflective engagement, critical analysis, and emotional openness. Thoughtful literary selection—combined with scaffolding activities that contextualize and personalize reading experiences—can help students build empathy for fictional characters and real-world cultural others. Finally, future research should continue to investigate the pedagogical and psychological conditions under which literature fosters empathy most effectively, with particular attention to genre, narrative structure, and individual reader differences (Lapshina et al., 2024).

## Conclusion

This study demonstrates the significant role of exposure to foreign literature in shaping cultural empathy and improving attitudes toward out-groups among Chinese readers. By examining direct and indirect pathways, the findings provide empirical support for literature as a medium of indirect intergroup contact that fosters emotional resonance, cognitive openness, and intercultural understanding (Moyer-Gusé et al., 2019; Oľhová et al., 2022). The tested sequential mediation model confirms that emotional investment and cultural empathy are key mechanisms through which foreign literature influences social perception. Readers who engage emotionally with diverse fictional narratives tend to develop greater empathy, which generalizes to real-world intergroup contexts.

These results contribute to a growing body of scholarship that highlights literature’s capacity to reduce prejudice, challenge stereotypes, and cultivate cross-cultural sensitivity (Li et al., 2019). Importantly, the study was conducted in a non-Western context, offering novel insights into how literary engagement operates within Chinese sociocultural and educational frameworks. This strengthens the case for the global relevance of literature as a tool for social change.

At the same time, the findings underscore the need for deliberate text selection and pedagogical mediation. Not all literary texts evoke empathy; some may unintentionally reinforce existing biases. Teachers are critical in selecting culturally rich, nuanced narratives and designing instructional practices that promote critical reflection and emotional engagement (Abdullayeva, 2024). Integrating such texts into educational settings can enhance intercultural competence and support the development of inclusive mindsets (Kaneeva et al., 2021).

Future research should examine the moderating role of individual differences, such as prior attitudes, genre preferences, or language proficiency, in shaping the empathic impact of literary exposure. It would also be valuable to investigate which narrative elements—such as voice, structure, or perspective—elicit sustained empathy and openness most effectively. Additionally, cross-cultural comparative studies could further clarify how national and educational contexts influence the reception and interpretation of foreign literature (Lütge et al., 2019).

In an increasingly interconnected world, cultivating empathy through literature remains a powerful and accessible strategy for promoting intercultural understanding. This study affirms that foreign literature does not merely enrich readers’ intellectual and emotional experiences and holds promise as a transformative medium for fostering social cohesion and a more empathetic global citizenry.

## Data Availability

The raw data supporting the conclusions of this article will be made available by the authors, without undue reservation.
